# confFuse: High-Confidence Fusion Gene Detection across Tumor Entities

**DOI:** 10.3389/fgene.2017.00137

**Published:** 2017-09-29

**Authors:** Zhiqin Huang, David T. W. Jones, Yonghe Wu, Peter Lichter, Marc Zapatka

**Affiliations:** ^1^Division of Molecular Genetics, German Cancer Research Center, Heidelberg, Germany; ^2^Division of Pediatric Neurooncology, German Cancer Research Center, Heidelberg, Germany; ^3^Hopp-Children's Cancer Center at the NCT Heidelberg, Heidelberg, Germany; ^4^DKFZ-Heidelberg Center for Personalized Oncology (DKFZ-HIPO), Heidelberg, Germany

**Keywords:** RNA-seq, next-generation sequencing, fusion gene, biomarkers, bioinformatics

## Abstract

**Background:** Fusion genes play an important role in the tumorigenesis of many cancers. Next-generation sequencing (NGS) technologies have been successfully applied in fusion gene detection for the last several years, and a number of NGS-based tools have been developed for identifying fusion genes during this period. Most fusion gene detection tools based on RNA-seq data report a large number of candidates (mostly false positives), making it hard to prioritize candidates for experimental validation and further analysis. Selection of reliable fusion genes for downstream analysis becomes very important in cancer research. We therefore developed confFuse, a scoring algorithm to reliably select high-confidence fusion genes which are likely to be biologically relevant.

**Results:** confFuse takes multiple parameters into account in order to assign each fusion candidate a confidence score, of which score ≥8 indicates high-confidence fusion gene predictions. These parameters were manually curated based on our experience and on certain structural motifs of fusion genes. Compared with alternative tools, based on 96 published RNA-seq samples from different tumor entities, our method can significantly reduce the number of fusion candidates (301 high-confidence from 8,083 total predicted fusion genes) and keep high detection accuracy (recovery rate 85.7%). Validation of 18 novel, high-confidence fusions detected in three breast tumor samples resulted in a 100% validation rate.

**Conclusions:** confFuse is a novel downstream filtering method that allows selection of highly reliable fusion gene candidates for further downstream analysis and experimental validations. confFuse is available at https://github.com/Zhiqin-HUANG/confFuse.

## Introduction

A fusion gene is typically generated from two different genes due to genomic aberrations, or rarely at the transcript level (e.g., read-through co-transcript events). It can lead to enhanced expression or altered activity of an oncogene, or deregulation of a tumor suppressor gene (Abate et al., 2014). Several technologies such as chromosome banding analysis and fluorescence *in situ* hybridization (FISH) have been successfully applied in detection of chromosomal alterations in the past (reviewed in e.g. Mertens et al., 2015). More recently, next-generation sequencing (NGS) technologies such as paired-end RNA-seq have enabled the generation of accurate, high-resolution data in a single experiment, allowing for unbiased genome-wide fusion detection (Steidl et al., 2011; Seshagiri et al., 2012; Chmielecki et al., 2013; Weischenfeldt et al., 2013; Lilljebjörn et al., 2014). A great number of fusion gene detection tools/pipelines have been developed to interrogate data from NGS, particularly paired-end RNA-seq (Carrara et al., 2013; Kumar et al., 2016). The performance of the tools differs in terms of sensitivity and specificity, depending on the individual algorithms and filtering methods applied (Kumar et al., 2016). Each of these tools/pipelines has its own advantages and weaknesses. A tool/pipeline should be properly chosen for each user's requirements, since one single tool/pipeline may not work best for all different data sets.

Fusion gene detection tools/pipelines generally consist of three major parts: firstly, mapping genomic data on reference genome/transcriptome based on existing alignment tools such as Bowtie (Langmead et al., 2009; Langmead and Salzberg, 2012) and BWA (Li and Durbin, 2009); second, individual methods for generating fusion candidates such as deFuse (McPherson et al., 2011), FusionMap (Ge et al., 2011), and SOAPfuse (Jia et al., 2013); and third, additional filtering algorithms to remove false positive candidates. The sensitivity of fusion gene detection mainly depends on the mapping ability in the alignment step and the specificity mostly depends on the methods of generating fusion candidates and the individual filtering methods.

Most of those tools/pipelines generate a large number of putative fusion transcripts even after filtering, of which most are likely to be false positives or of low biological interest (e.g., precursor read-through transcripts), making it hard to prioritize candidates for experimental validation. Additional filtering methods were developed based on individual datasets in order to select reliable candidates (Cancer Genome Atlas Research Network, 2013; Torres-García et al., 2014). Those individual filters of fusion gene candidates, however, may have a bias toward cancer or cell type-specific artifacts. A method which can work across different data sets would be very helpful for users. Some false positive fusion predictions may be due to sequencing/alignment artifacts or sequencing library preparation (Mertens et al., 2015). Furthermore, strict filtering can decrease sensitivity of true fusion detection (Torres-García et al., 2014). Therefore, we developed confFuse, a new scoring algorithm, which can be applied on paired-end RNA-seq across tumor entities with both high sensitivity and high detection accuracy.

## Materials and methods

confFuse was designed to rank fusion candidates based on deFuse output by assigning each fusion candidate a confidence score, with the aim of markedly reducing the total number of fusion candidates while retaining a high recall rate for true positives. It takes multiple features into account, including some from the standard deFuse output and also newly generated features, with each given a specific score weight. These features are closely relevant to mapping performance and fusion-related structure. The final confidence score is the sum of the score weights of different single/combined features (the initial baseline score is 10). These parameter weightings were manually optimized in comparison to a known validated fusion list, in order to achieve a balance between eliminating false positives whilst retaining true fusions. Fusion candidates scoring between 8 and 10 are considered as being high-confidence candidates. The main features used to calculate these score weights are described below and summarized in Table S1.

### Training data

Sixteen recently published pediatric glioblastoma RNA-seq samples were chosen as the first training data (International Cancer Genome Consortium PedBrain Tumor Project, 2016). Fusion gene candidates in these 16 samples were first identified by tools SOAPfuse and TopHat2-Fusion (Kim et al., 2013). High-confidence candidates were then filtered for common artifacts and by visual inspection of fusion break points of exons between two fusion partners (International Cancer Genome Consortium PedBrain Tumor Project, 2016). In total, 40 fusion genes were successfully verified by RT-PCR among the 16 samples. The first training data was mainly used to identify and select features from deFuse reports.

The second training data contains 96 RNA-seq samples from seven studies, including pilocytic astrocytoma (*n* = 7; Jones et al., 2013), thyroid cancer (*n* = 5; Ricarte-Filho et al., 2013), glioblastoma (*n* = 47; Bao et al., 2014), lung adenocarcinoma (*n* = 28; Seo et al., 2012), ependymoma (*n* = 7; Pajtler et al., 2015), lung cancer liver metastasis (*n* = 1; Ju et al., 2012), and biphenotypic sinonasal sarcoma (*n* = 1; Wang et al., 2014). Those samples, including 126 experimentally verified fusion genes, were used to optimize the score weights of varied features.

### Validation data

A published study of early-onset prostate cancer including 11 RNA-seq samples were chosen for validation *in silico* (Weischenfeldt et al., 2013) and three primary breast cancer samples were used for experimental validation.

### Artifact list

**Figure 1:**
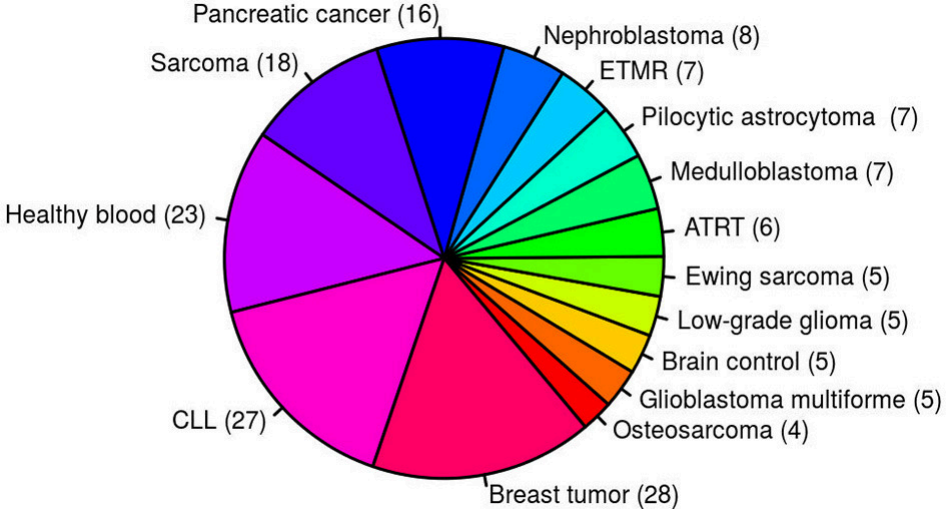
One hundred and seventy-one paired–end RNA–seq samples from 15 different entities were used to generate an artifact list of fusion genes. ETMR, embryonal tumor with multilayered rosettes; CLL, chronic lymphocytic leukemia; ATRT, atypical teratoid rhabdoid tumor.

Despite a prominent role for oncogenic gene fusions in multiple cancer types, it is relatively rare for the exact same fusion to be detected across multiple, unrelated tumor entities. Fusions identified in multiple samples from different tumor entities based on currently available fusion detection tools are therefore mostly considered to be of high false positive rate. This high false positive prediction may be due to genomic complexity such as repeat regions or mapping artifacts in the alignment step. In total, 171 paired-end RNA-seq samples from 15 different entities were used to generate an artifact list of fusions identified in multiple samples from several different entities (Figure 1). To increase the sensitivity, a small number of verified fusions were manually excluded from the artifact list. We aim to assign high-confidence fusion genes a score between 8 and 10, and consider fusions contained in the artifact list to be of high false positive rate. confFuse therefore assigns a negative score (−6) to those fusions in the artifact list in order to rank them outside of the range of confident predictions.

**Figure 2:**
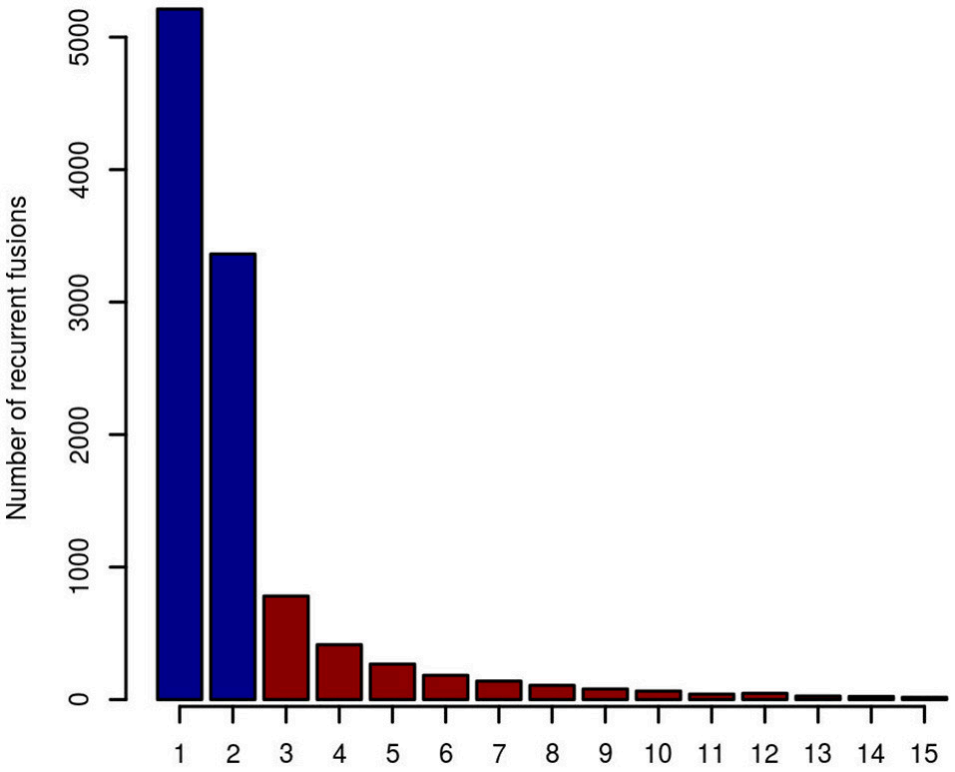
The number of recurrent fusion genes in 15 different entities. Fusions identified in more than two entities were selected for the final artifact list, resulting in 2,190 artifact fusions labeled with red color.

When taking fusion candidates identified by deFuse (version v0.6.1) in no less than three entities (recurrence ≥ 3), 2190 fusions were included in the artifact list (Figure 2). For candidates identified in ≥4 and ≥5 entities, there are 1,409 and 995 fusions in the artifact list, respectively. In this study, we chose a threshold of three entities for the final artifact list. Of note, a small number of additional putative artifacts are still identified with each increase in the number of different tumor types, suggesting that accuracy could be further improved by increasing the complexity of the data set used to generate the artifact list.

In total, 64% (5881/9169) of putative fusion transcripts (*n* = 9169) identified in the second training data were found in the artifact list (*n* = 2190). Among them, 62.3% (3666/5881) were fusions from adjacent genes and 91.5% (5378/5881) were identified by deFuse as likely being a product of alternative splicing.

### Split reads and spanning reads

**Figure 3:**
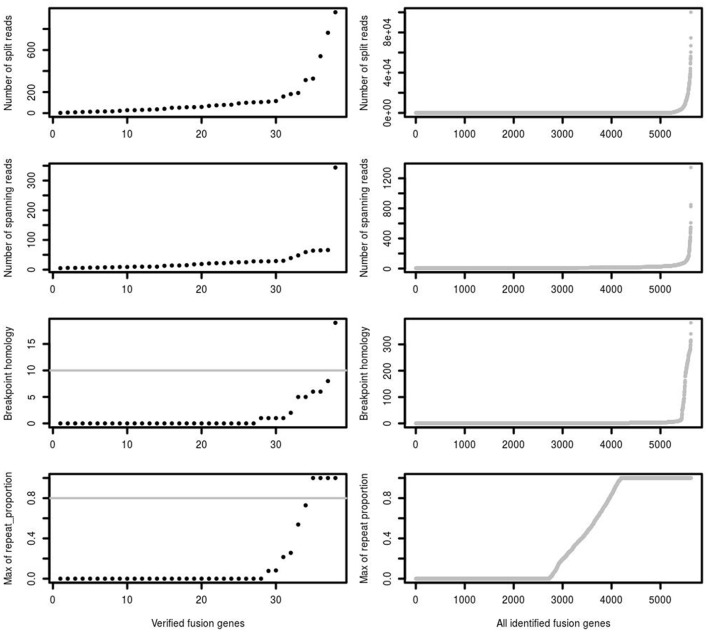
The number of split reads, spanning reads and breakpoint homology between verified and identified fusions. deFuse reported most of the verified fusion genes in 16 glioblastoma samples as containing <200 split reads, <50 spanning reads and <10 bp breakpoint homology. The maximum proportion of spanning reads in fusion partners aligned on a repeat region (repeat_proportion) is <80% among most of the verified fusions.

One of the most important features supporting a true fusion event is the number of split reads and spanning reads. Since this is related not just to mapping performance, but also to fusion gene expression levels and sequencing depth, we found that setting a simple threshold on the number of split and spanning reads could not best distinguish true and false positive predictions. For example, a true fusion gene with low expression and low coverage sequencing depth may have only a few detectable split and spanning reads. A false positive fusion gene may have a large number of reads due to mapping artifacts and/or unreliable reads aligned to multiple genomic locations. Comparing verified fusions with all initial calls, the distribution of number of split reads and spanning reads between them is similar, of which the majority are of low read numbers (Figure 3). Most of the verified fusions in the first training data have <200 split reads and 50 spanning reads. A threshold purely on the number of split and spanning reads therefore cannot distinguish true and false positive fusion predictions.

In addition, the mapping quality of reads should also be considered. Some spanning reads can be aligned to more than one genomic position, indicating reads of low mapping quality which do not reliably support a fusion event. Breakpoint homology is the number of nucleotides near the fusion break point which can map equally well to both fusion partners, with very high breakpoint homology therefore suggesting more ambiguous support for a fusion event. Most of the verified fusions contain <10 homologous bases at the fusion breakpoint (Figure 3). confFuse therefore assigns a negative score (−1) when breakpoint homology is ≥10. If spanning reads are mapped on a repeat region, it is difficult to identify where they are originally from, and thus those spanning reads are considered as low mapping quality. Therefore, confFuse assigns a negative score to those fusions with the majority of spanning reads aligned on repeat regions, e.g., −0.5 score for fusions of 80% up to 90% of spanning reads aligned on a repeat region and −1 score for those fusions of >90% (Figure 3; Table S1).

Fusion genes with different fusion transcripts (i.e., splice variants) in the same sample may be of high true positive rate, especially those fusion transcripts with a high count of split reads and spanning reads. We observed that deFuse sometimes reports multiple fusion transcripts for the same pair of fusion partner genes (occurrence of the same fusion pairs) indicating high probability of true fusion events. confFuse thus considers the multiple fusion transcripts from a fusion gene as a positive factor supporting a true fusion event.

By combining with the occurrence of the same fusion pairs, the number of supporting reads, mapping quality of reads, possible mapping artifacts and other fusion structure related features mentioned below, confFuse also assigns a positive score from 0.5 to 2.5 to fusions with a high number of split and uniquely mapped spanning reads or a negative score from −0.5 to −2.0 otherwise, such as −1.5 score when all the spanning reads are mapped on more than one genomic location (Table S1).

### Fusion structure related features

**Figure 4:**
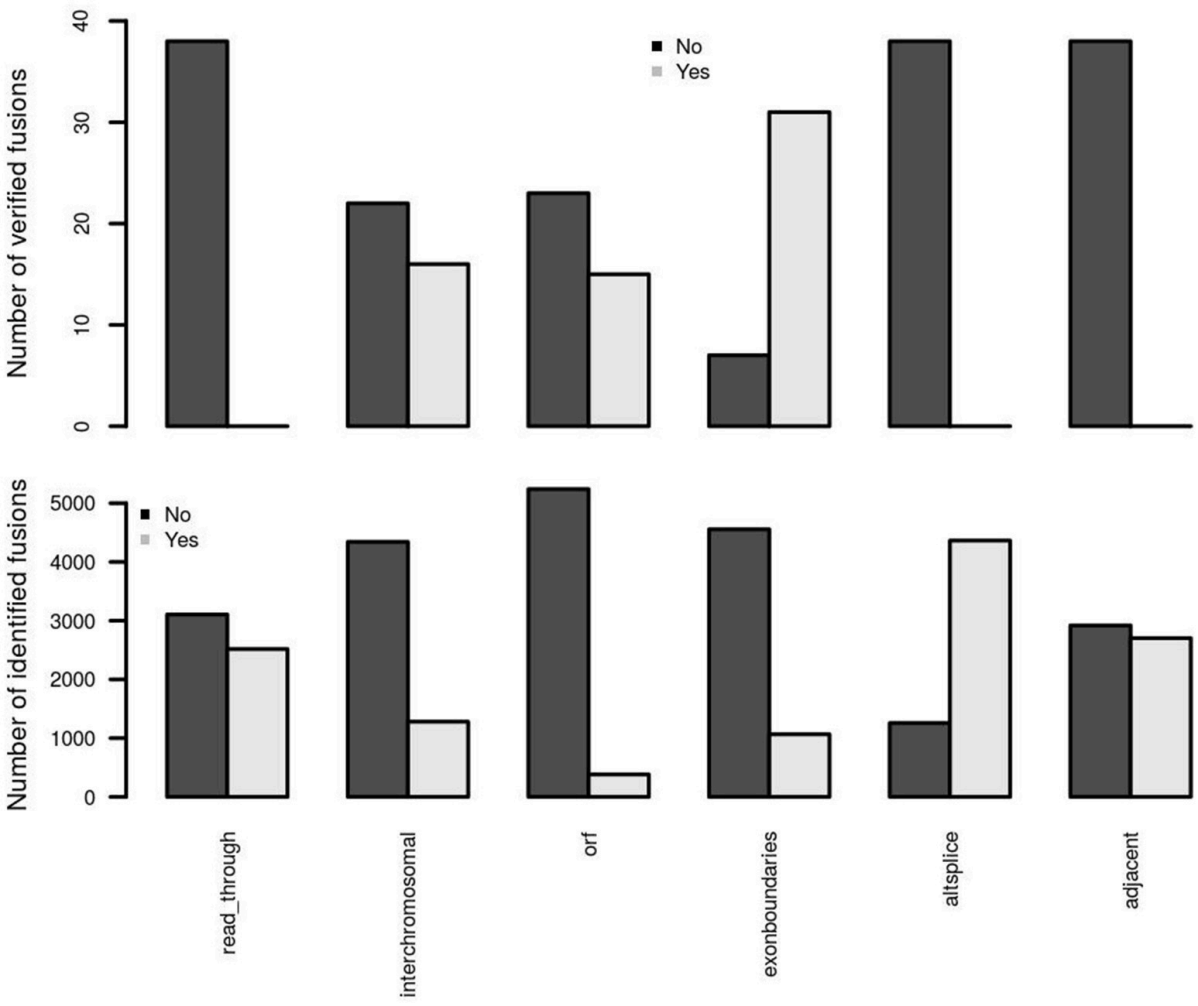
Important feature distributions between verified and identified fusions. In the first training dataset, the verified fusions (*n* = 38) do not have any feature of read-through, alternative splicing and adjacent genes (which may also be partly due to a selection bias in those fusions selected for verification). Comparing with identified fusions, higher ratios of interchromosomal, open reading frame (orf) and exon boundaries in verified fusions were detected.

Two adjacent genes in the same orientation may give rise to an apparent fusion due to read-through transcription or aberrant splicing rather than genomic rearrangement. Although some may acquire novel function, the vast majority are expected to be false positives in terms of their biological relevance. deFuse reports an altsplice feature, indicating that a fusion may arise from alternative splicing between adjacent genes. In the first training data, verified fusion genes do not contain any read-through or alternative splicing events (Figure 4). More than 75% of initially identified fusions are, however, from an alternative splicing event. Therefore, confFuse takes those fusions with read-through or alternative splicing as high false positive fusion candidates by assigning a negative score (−4) in order to separate them from high confidence candidates. An adjacent gene fusion ESR1:CCDC170 was reported from 22 of 990 tumor samples (Veeraraghavan et al., 2014), showing the possibility of true fusions from adjacent genes. Fusion candidates from adjacent genes but without a read-through or altsplice tag are therefore given a reduced penalty of only −0.5. Furthermore, a higher ratio of inter- rather than intra-chromosomal fusions were detected in the verified fusions comparing with all identified fusions (Figure 4), and confFuse therefore also assigns a modest negative score (−0.5) for intrachromosomal predictions (users can reset the score weight to zero if distance between fusion gene partners is large).

True oncogenic fusions typically preserve the open reading frame in order to form a functional fusion protein, and the precise location of a fusion breakpoint point plays a critical role in demonstrating evidence supporting true positive fusions. When the location of a fusion splicing point is at a known exon boundary, such a fusion is more likely to be a true positive. Comparing with all identified fusions, we observed higher ratios of verified fusions preserving an open reading frame and showing fusion splicing points at exon boundaries (Figure 4). confFuse assigns a negative score to fusions with non-detected open reading frame (−1 score) and with splicing point not at an exon boundary (−1.5 score). It is also more likely to be of low biological interest when a break point is located downstream of the 3′ fusion partner because such predictive fusions may not have biological function or may arise from mapping artifacts. confFuse takes those fusions as low-confidence ones by assigning a negative score (−4).

## Results and discussion

### Recovery rate of verified fusions

**Figure 5:**
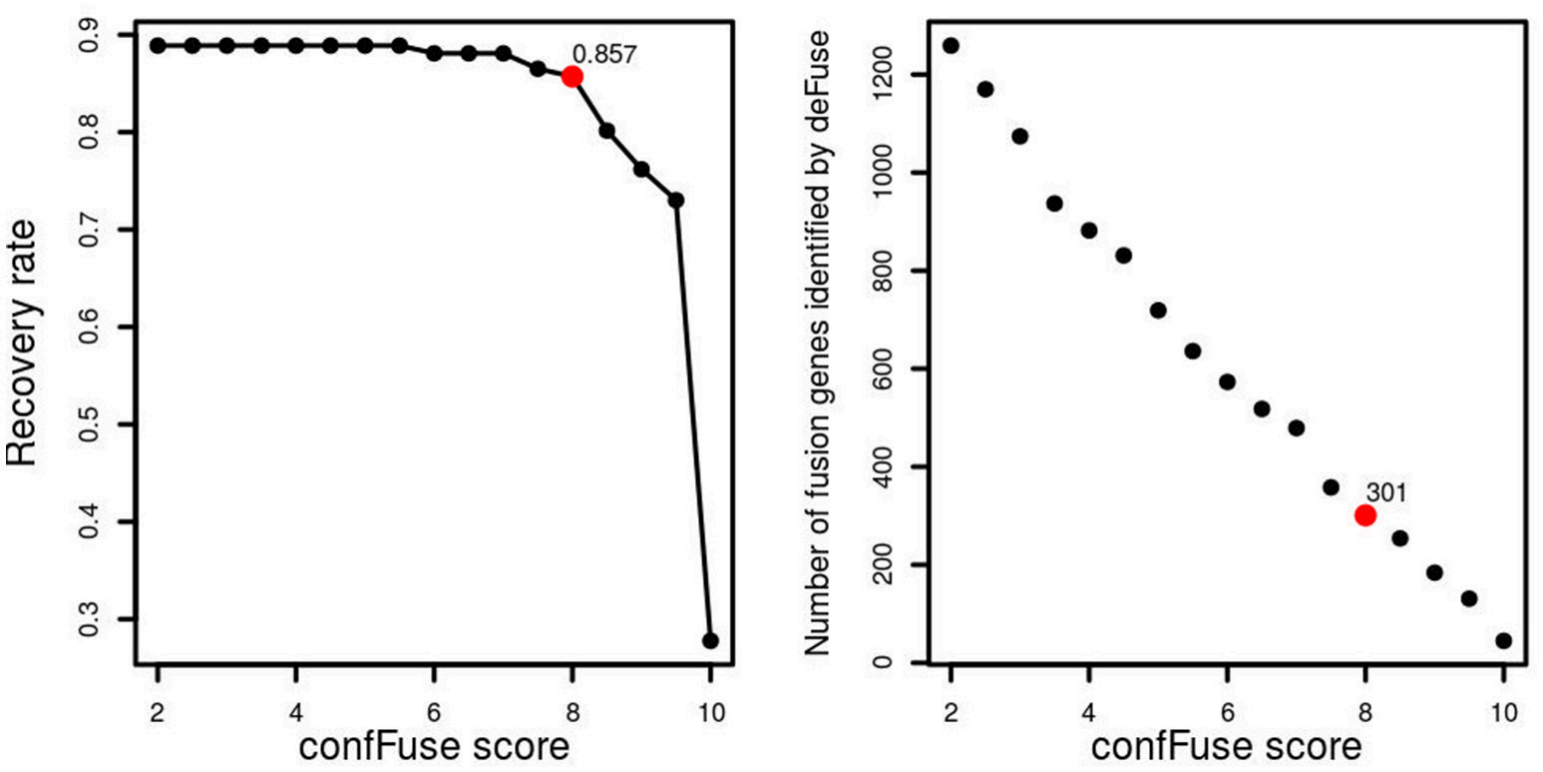
confFuse score and recovery rate in 96 published samples. In total, 126 fusion genes were validated, 113 of which were identified by deFuse. confFuse detected 108 of 126 known validated fusions with score threshold 8. The right-hand figure shows the correlation between confFuse score and the number of fusions identified by deFuse.

In the first training data (*n* = 16), 77.5% (31/40) of verified fusions were scored ≥8 by confFuse, 15% (6/40) is of 6 ≤ score <8, and 2.5% (1/40) is <6 (Table S2). In total, 8,083 fusion gene candidates (9,169 putative transcripts) from the second training data (*n* = 96) were identified by deFuse using default settings, of which 126 fusions were previously validated by RT-PCR (Table S3). confFuse called 301 high-confidence fusion genes (score ≥ 8, 301/8,083, 3.7%). Among the 301 fusions were 108 of the 126 validated fusions, resulting in a recovery rate of 85.7% (108/126). The remaining previously validated fusions were either scored <8 (*n* = 5) or were not detected or were already filtered by default deFuse parameters prior to application of confFuse (*n* = 13; Table S4). The correlation between recovery rate and confFuse score in the second training data is given in Figure 5. As annotation features such as read-through are not provided by fusionMap and soapFuse, the number of supporting reads was chosen to compare recovery rate. As the threshold for the required minimum number of supporting reads increases, the recovery rate by soapFuse and fusionMap decreases (Figure S1, Tables S9, S10). For fusions with more than five supporting reads, fusionMap predicted 964 fusions, of which 101 were previously verified, resulting in a recovery rate ~80% (101/126); soapFuse predicted 562 fusions, of which 65 were verified before, resulting in a recovery rate ~51.6% (65/126). Thus, setting a threshold only on the number of supporting reads cannot retain a high recovery rate and results in decreasing sensitivity.

### Comparison of defuse probability and confFuse confidence score

**Figure 6:**
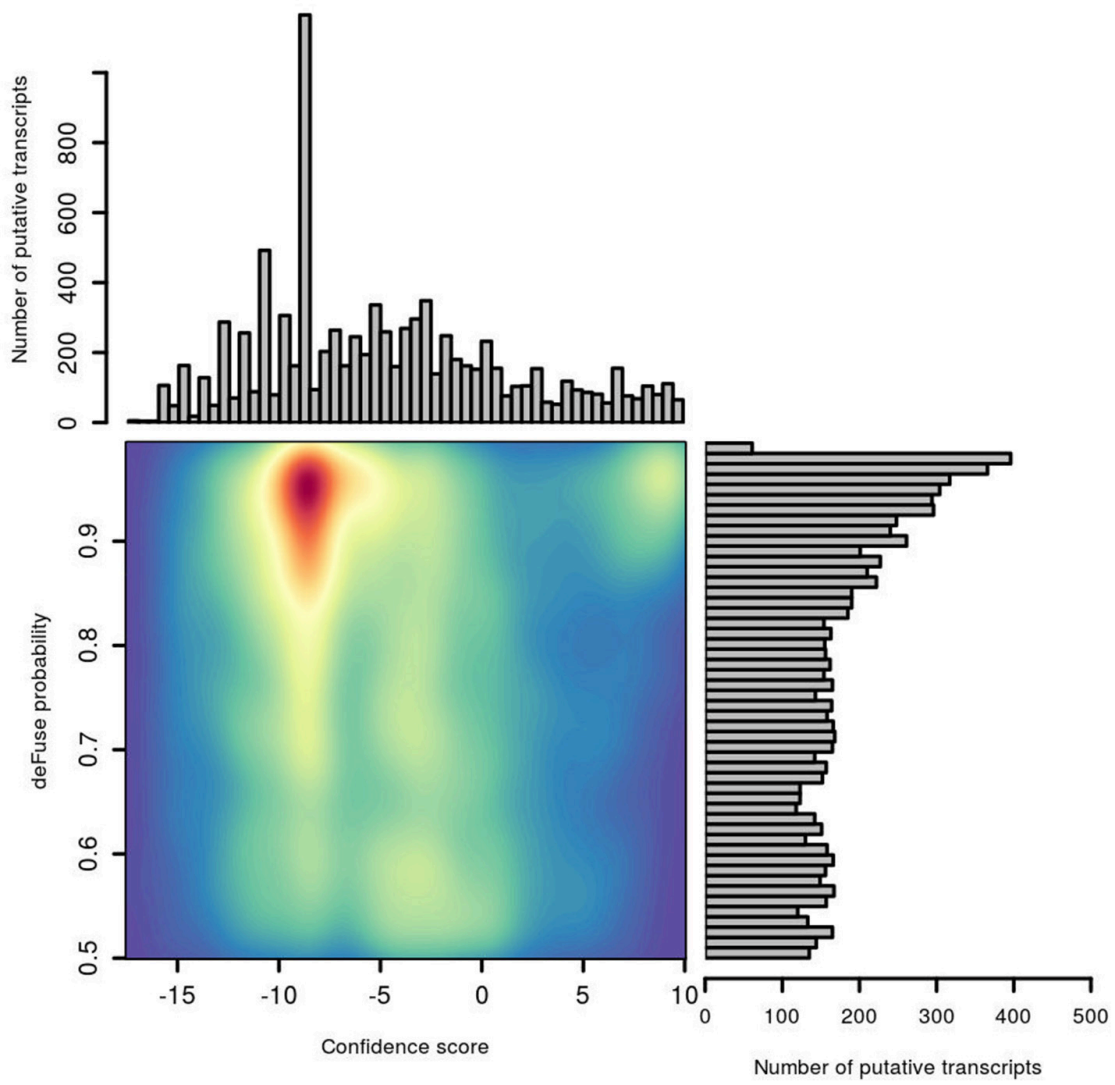
Comparison of probability predicted by deFuse and confidence score by confFuse in 96 published RNA-seq samples. High density is of red color and low density is of blue color.

The distribution of deFuse's own probability score was compared with our confFuse confidence score for all putative fusion transcripts in the second training data (Figure 6). Notably, there are many putative transcripts with a high deFuse probability which were assigned a low score (~ −8) by confFuse, of which most are in the artifact list or of alternative splicing feature. None of these are in the list of 126 known validated fusions in the second training data. Most of the verified fusions (108/126) are located in the range of confFuse confidence score no less than eight. It demonstrates that confFuse is able to identify the verified fusions among hundreds of putative fusions with high deFuse probability.

### Comparison of alternative fusion detection tools

**Figure 7:**
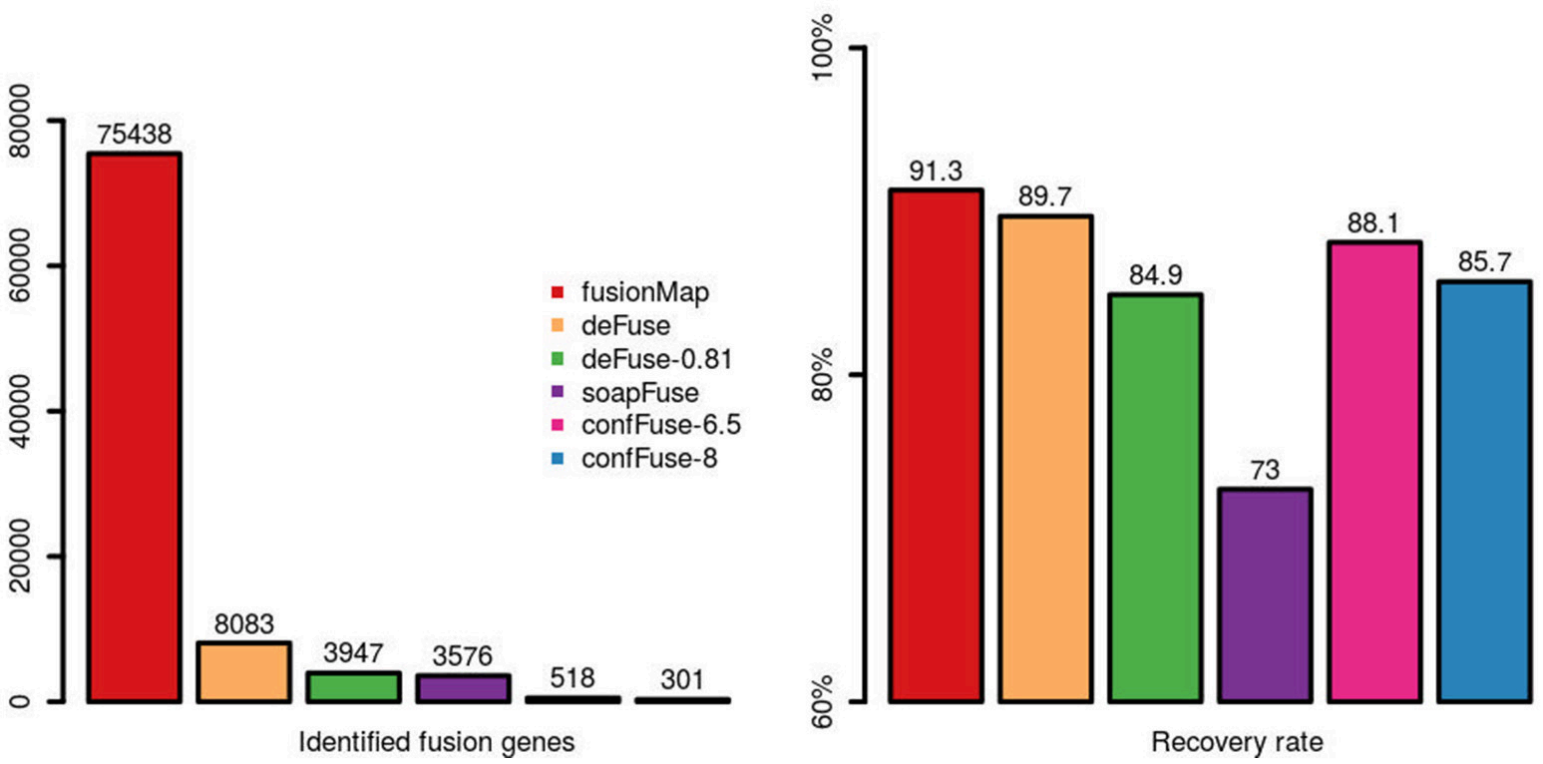
Identified fusion genes and recovery rate of validated fusions among different tools. One hundred and twenty-six fusions were previously validated by RT-PCR. Five methods (fusionMap, deFuse, deFuse-0.81, confFuse-6.5, and confFuse-8) performed similarly in terms of recovery rate. confFuse generated much less fusion candidates than the others (higher specificity) while identifying comparable number of validated fusions (similar sensitivity).

Comparing across tools, fusionMap, deFuse (default probability score threshold = 0.5), deFuse-0.81 (deFuse with probability score threshold set to ≥0.81, as used in McPherson et al., 2011), soapFuse, confFuse-6.5 (score ≥ 6.5) and confFuse-8 (high-confidence candidates scored ≥ 8) showed recovery rates of 91.3, 89.7, 84.9, 73, 88.1, and 85.7% respectively for the 126 validated fusions (Figure 7; Tables S4–S6, S9, S10), indicating confFuse can dramatically reduce the number of candidates (from 8,083 to only 301) without compromising detection accuracy when compared with other available tools.

### Validation of confFuse predicted candidates

**Figure 8:**
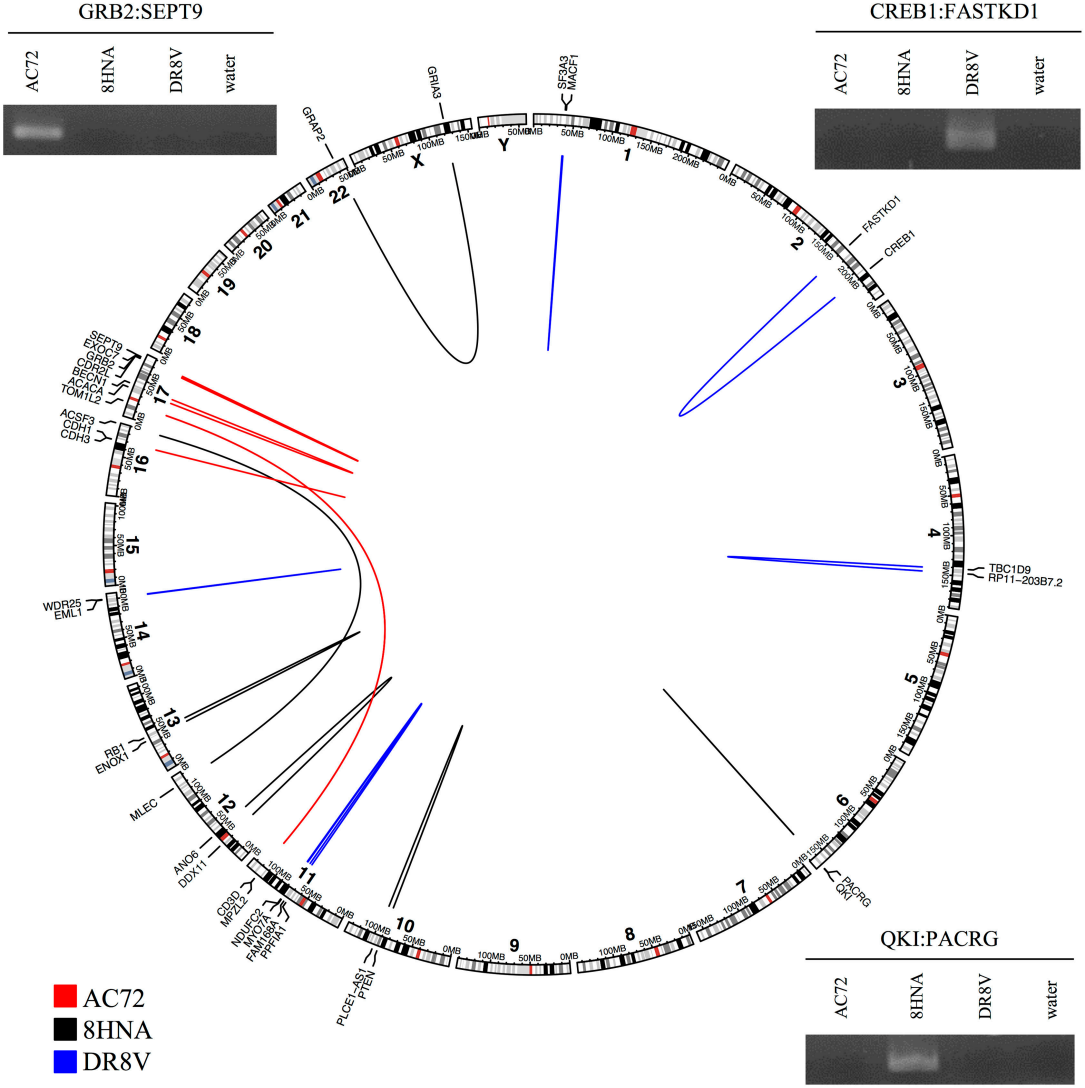
Eighteen high-confidence fusions validated by RT-PCR followed by Sanger sequencing in three primary breast tumor samples. Circular layout is based on tool (Gu et al., 2014).

To evaluate the accuracy of high-confidence candidate predictions (score ≥ 8), three primary breast tumor samples were sequenced to generate paired-end RNA-seq data. In total, deFuse predicted 1,026 fusion genes in the three samples, of which 18 scored ≥8 by confFuse. All 18 high-confidence candidates were validated with RT-PCR followed by Sanger sequencing, resulting in 100% validation rate (Figure 8; Table S7). To the best of our knowledge, the 18 novel fusion genes haven't been validated before. Interestingly, one of them (QKI:PACRG) was predicted in three of 1,019 breast cancer samples from TCGA (www.tumorfusions.org), indicating that QKI:PACRG may be a novel recurrent fusion in breast cancer. QKI can suppress cell proliferation and prevent inappropriate activation of the Notch signaling pathway in lung cancer (Zong et al., 2014) and PACRG is an evolutionarily conserved protein with currently unclear function (Dawe et al., 2005). Furthermore, we randomly chose some candidates scored <8 for validation. Nine of 13 candidates (6.5 ≤ score ≤ 7.5, medium confidence) and three of 10 candidates (0.5 ≤ score ≤ 6, low confidence) were experimentally verified (Table S7). Most of the genes involved in verified fusions were in the expression level of FPKM <50 (median≈6.44; Figure S2), indicating that it is not only very highly-expressed candidates being detected but also lowly-expressed ones.

To find out whether or not those verified fusions are individual tumor specific rather than simply artifacts of pan-breast tumor expression, the primers of verified fusions in each sample were used for validation in the other two breast primary tumor samples as control. In total, primers (Untergasser et al., 2012) of 14 verified fusions were used, of which 13 showed true negative in two control samples and one showed a false negative in one control sample DR8V (Figure S3). This “false negative” fusion (CD3D:TOM1L2) in sample DR8V shows a much weaker band in gel compared with the one in sample AC72 where the fusion was true positive, and appears as a double band of different size to AC72, possibly indicating an unspecific PCR product.

In addition, 11 published early-onset prostate cancer samples were used as an *in silico* validation dataset. Eight hundred and forty-nine fusion genes were called by deFuse, 24 of which were identified as high-confidence fusion (score ≥ 8) by confFuse. The well-known E26 transformation-specific (ETS) fusions were detected by confFuse in 10 of 11 samples, which are the same as previously published results (Weischenfeldt et al., 2013). Among the 24 fusions, 17 were confirmed by DNA-seq, FISH validation or known ETS fusions, resulting in a ~70% (17/24) recovery rate (Table S8).

Overall, confFuse can classify fusion candidates into three groups, namely high-confidence (8 ≤ score ≤ 10), medium-confidence (6.5 ≤ score ≤ 7.5) and low-confidence (score ≤ 6) fusions, which makes the users more convenient to prioritize candidates for validations. Not only novel and biologically relevant fusions can be identified by confFuse, but also well-known fusions across tumor entities can be detected by confFuse.

## Conclusions

Based on deFuse reports, the scoring algorithm confFuse assigns each putative fusion transcript a confidence score. In three breast tumor samples, we achieved 100% true positive rate for high-confidence fusion candidates. Once more verified fusion genes are available as reference data, score weight optimization could be further improved. Users can also customize the score weights based on their experience to better analyze their specific data. In summary, confFuse can reliably select high-confidence fusion genes that are more likely to be biologically relevant, achieving both high validation rate and high detection accuracy, while reducing the number of candidates to a realistic number for validation.

## Availability of data and material

Training dataset:

Pediatric glioblastoma: EGAS00001001139 (International Cancer Genome Consortium PedBrain Tumor Project, 2016)
Pilocytic astrocytoma: EGAS00001000381 (Jones et al., 2013)
Thyroid cancer: SRP027364 (Ricarte-Filho et al., 2013)
Glioblastomas: GSE48865 (Bao et al., 2014)
Lund adenocarcinoma: ERP001058 (Seo et al., 2012)
Ependymoma: Application from the authors (Pajtler et al., 2015)
Lung cancer liver metastasis: ERP001058 (Ju et al., 2012)
Biphenotypic sinonasal sarcoma: GSE52257 (Wang et al., 2014)

Validation dataset:

Prostate cancer: EGAS00001000258 (Weischenfeldt et al., 2013)

## Ethics statement

This study was carried out in accordance with the recommendations of the ethics committee of the University of Heidelberg, with written informed consent obtained from all subjects in accordance with the Declaration of Helsinki.

## Author contributions

ZH wrote the manuscript and implemented the method. PL, YW, DJ, ZH, and MZ designed the experimental validation and interpreted the results. ZH designed the primers and YW performed the validation. DJ, YW, and MZ revised the manuscript. All the authors read and approved the final manuscript.

### Conflict of interest statement

The authors declare that the research was conducted in the absence of any commercial or financial relationships that could be construed as a potential conflict of interest.

## Supplementary material

Table S1 The score weights of single and combined features in confFuse scoring algorithm.

Table S2 The verified fusion genes in the first training data.

Table S3 The verified fusion genes in the second training data.

Table S4 The recovery of verified fusions in deFuse, confFuse-6.5, and confFuse-8.

Table S5 The recovery of verified fusions in fusionMap.

Table S6 The recovery of verified fusions in soapFuse.

Table S7 The experimental validations in three breast tumor samples.

Table S8 The high-confidence fusions in prostate cancer samples.

Table S9 The correlation of verified fusions and supporting reads in fusionMap.

Table S10 The correlation of verified fusions and supporting reads in soapFuse.

## Abbreviations

FPKM: Fragments Per Kilobase of exon per Million mapped reads.
