# Supplementary material for: confFuse: High-Confidence Fusion Gene Detection across Tumor Entities
Source: Front Genet. 2017 Sep 29;8:137. doi: 10.3389/fgene.2017.00137 (PMC5627533; doi:10.3389/fgene.2017.00137)
Supplement: Supplementary file 2 [file Image1.PDF]

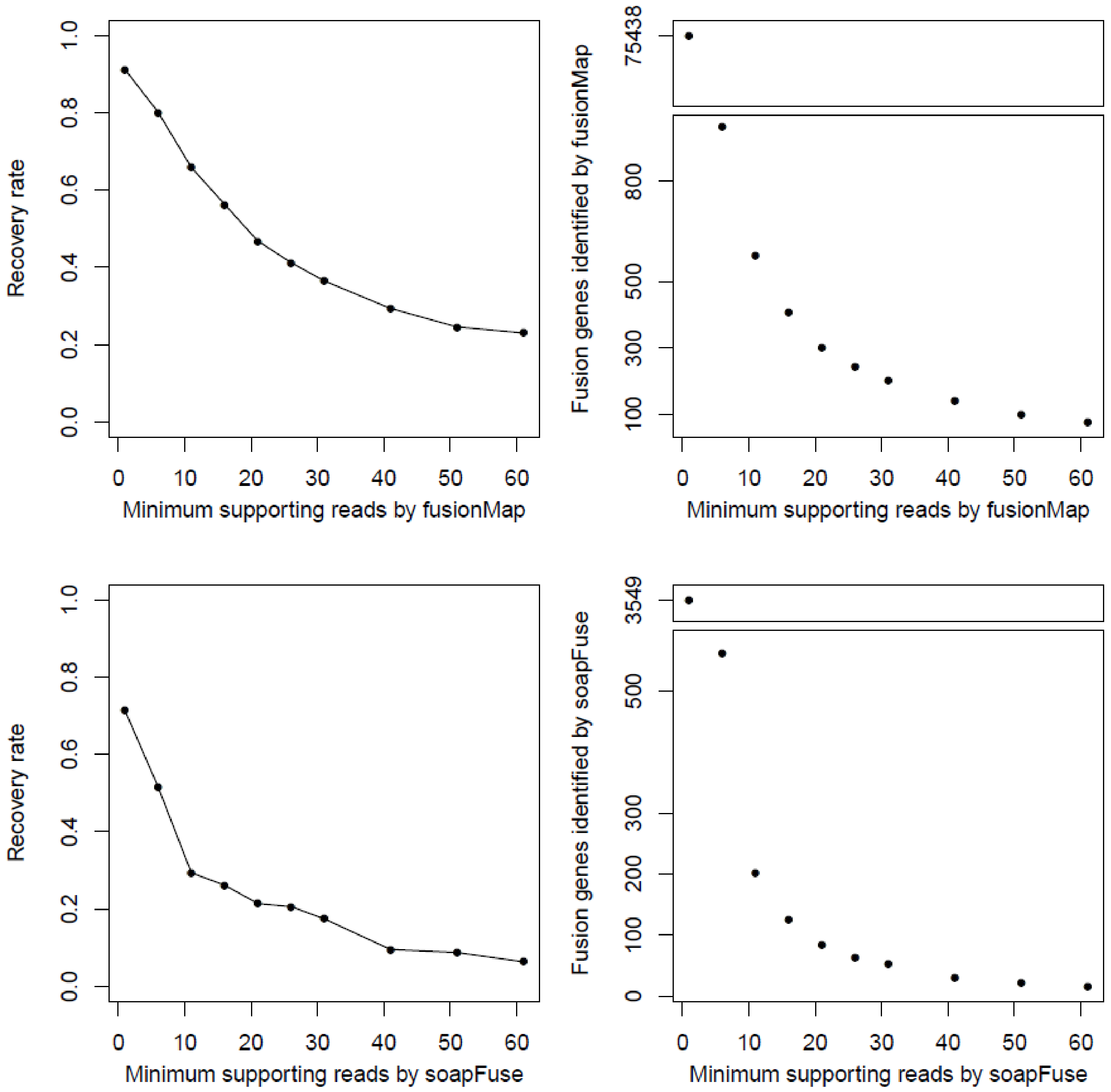

Figure S1: The correlation of recovery rate and minimum supporting reads in fusions identified in fusionMap and soapFuse. The thresholds are 1, 6, 11, 21, 26, 31, 41, 51 and 61.

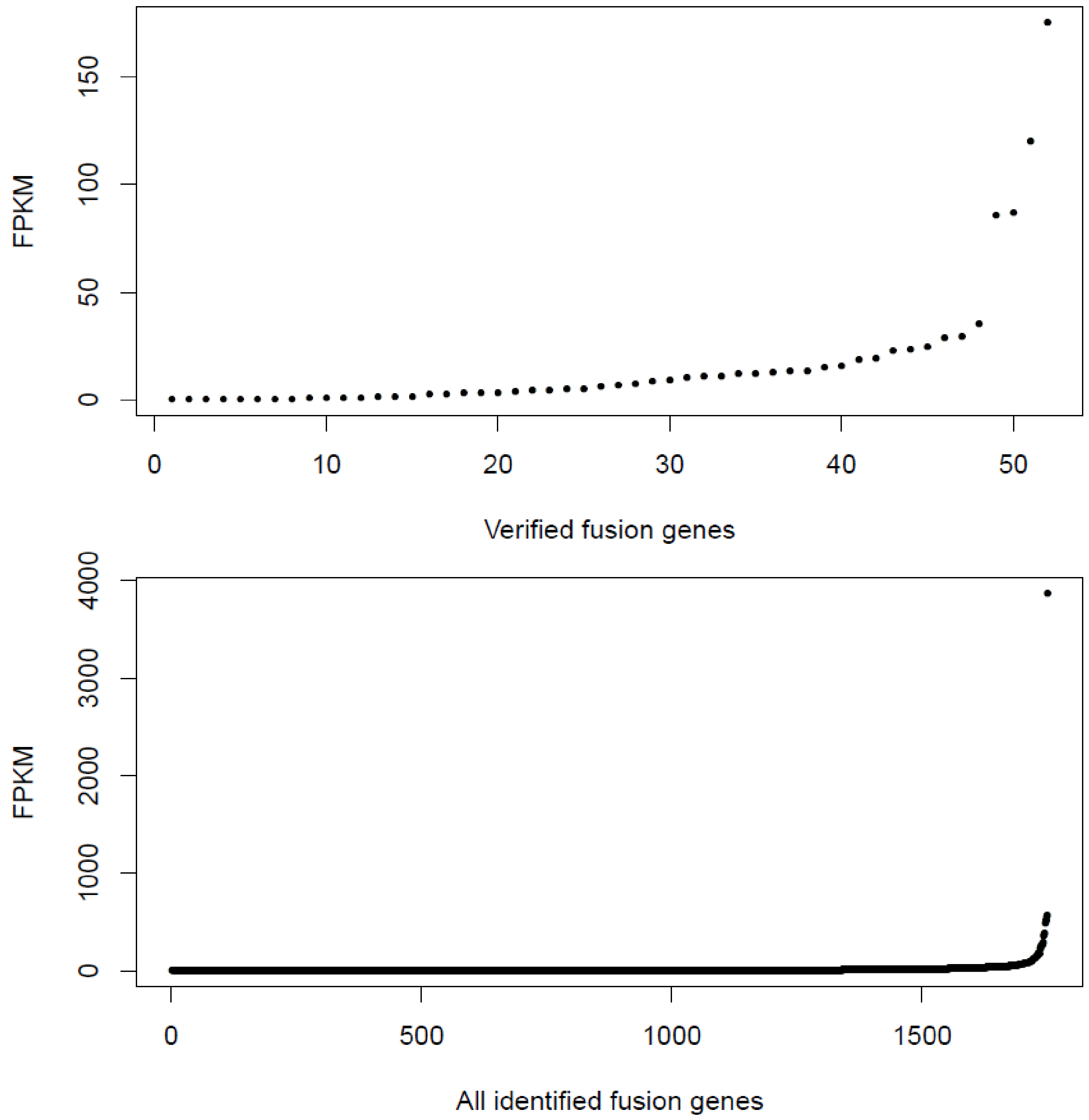

Figure S2: The expression of genes involved in verified fusions in three breast tumor samples. The bottom figure shows the expression of all identified fusion partner genes.

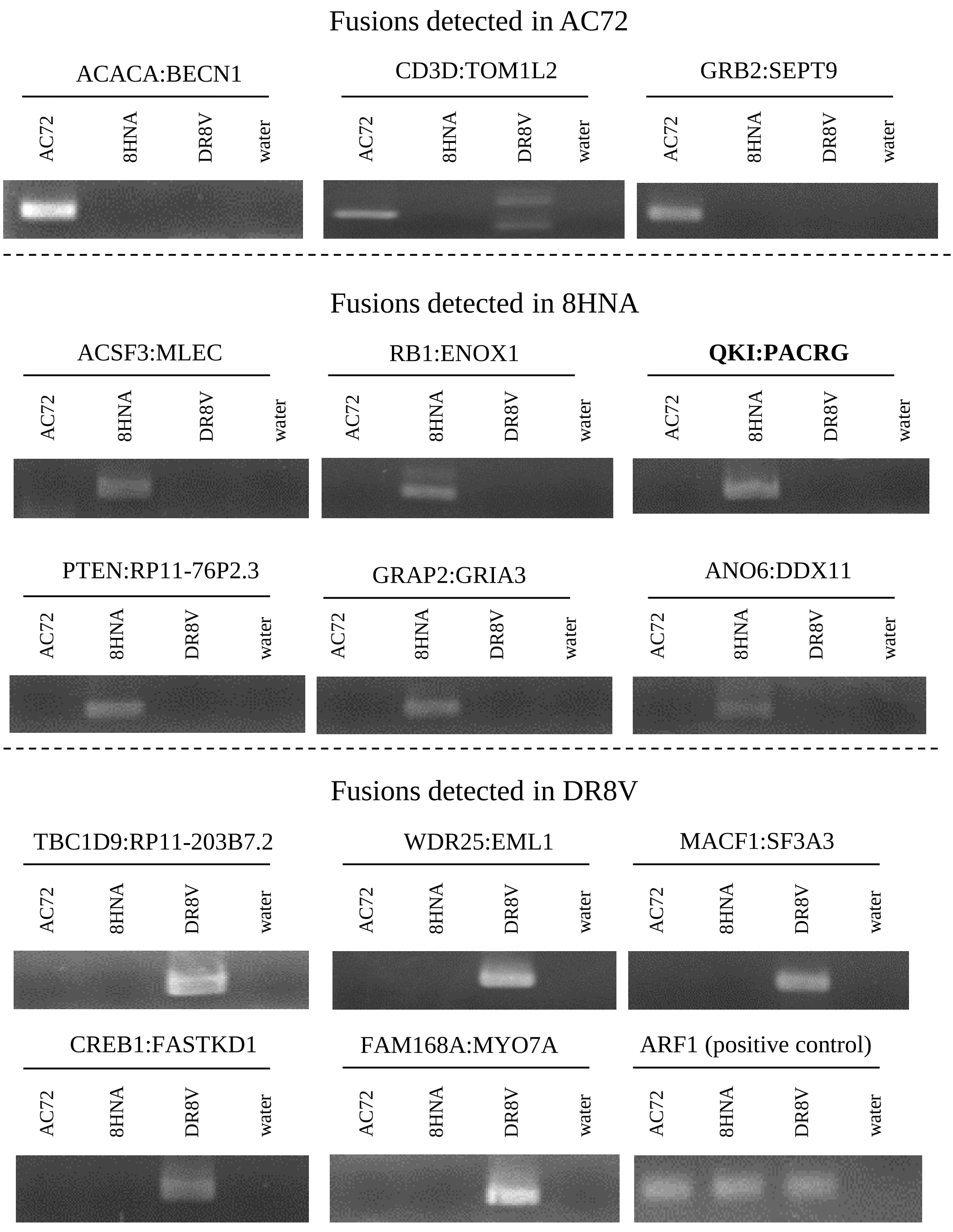

Figure S3: Validations of 14 paired primers in three breast cancer samples.
